# Phase II Window Study of Olaparib Alone or with Cisplatin or Durvalumab in Operable Head and Neck Cancer

**DOI:** 10.1158/2767-9764.CRC-23-0051

**Published:** 2023-08-10

**Authors:** Myrto Moutafi, Georgia-Angeliki Koliou, George Papaxoinis, Panagiota Economopoulou, Ioannis Kotsantis, Maria Gkotzamanidou, Maria Anastasiou, Dimitrios Pectasides, Efthymios Kyrodimos, Alexander Delides, Evangelos Giotakis, Nikolaos G. Papadimitriou, Ioannis G. Panayiotides, Christos Perisanidis, Aileen I. Fernandez, Vasiliki Xirou, Christos Poulios, Eleni Gagari, Vesal Yaghoobi, Niki Gavrielatou, Saba Shafi, Thazin Nwe Aung, Andromachi Kougioumtzopoulou, Vassilis Kouloulias, Konstantinos Palialexis, Stavros Gkolfinopoulos, Areti Strati, Evi Lianidou, George Fountzilas, David L. Rimm, Periklis G. Foukas, Amanda Psyrri

**Affiliations:** 1 Second Department of Internal Medicine, Medical Oncology Section, National and Kapodistrian University of Athens, Attikon University Hospital, Athens, Greece.; 2 Department of Pathology, Yale School of Medicine, New Haven, Connecticut.; 3 Yale Cancer Center, Yale School of Medicine, New Haven, Connecticut.; 4 Section of Biostatistics, Hellenic Cooperative Oncology Group, Data Office, Athens, Greece.; 5 Second Department of Internal Medicine, Agios Savvas Cancer Hospital, Athens, Greece.; 6 Second Department of Internal Medicine, Medical Oncology Section, Hippokration General Hospital, Athens, Greece.; 7 Department of Otolaryngology-Head and Neck Surgery, Hippokration General Hospital, University of Athens, Athens, Greece.; 8 Second Otolaryngology Department, National and Kapodistrian University of Athens, Attikon University Hospital, Athens, Greece.; 9 Second Department of Pathology, National and Kapodistrian University of Athens, Attikon University Hospital, Athens, Greece.; 10 Department of Oral and Maxillofacial Surgery, School of Dentistry, National and Kapodistrian University of Athens, Athens, Greece.; 11 Department of Pathology, Aristotle University of Thessaloniki, School of Health Sciences, Faculty of Medicine, Thessaloniki, Greece.; 12 Oral Medicine Clinics, A. Syggros Hospital of Dermatologic and Venereal Diseases, Department of Dermatology, School of Medicine, University of Athens, Athens, Greece.; 13 Second Department of Radiology, Radiotherapy Unit, National and Kapodistrian University of Athens, Attikon University Hospital, Athens, Greece.; 14 Department of Medical Oncology, Cyprus Oncology Centre, Nicosia, Cyprus.; 15 Analysis of Circulating Tumor Cells Lab, Lab of Analytical Chemistry, Department of Chemistry, National and Kapodistrian University of Athens, Athens, Greece.; 16 German Oncology Center, Limassol, Cyprus.; 17 Laboratory of Molecular Oncology, Hellenic Foundation for Cancer Research/Aristotle University of Thessaloniki, Thessaloniki, Greece.; 18 Aristotle University of Thessaloniki, Thessaloniki, Greece.

## Abstract

**Purpose:**

We conducted a phase II randomized noncomparative window of opportunity (WOO) trial to evaluate the inhibition of cellular proliferation and the modulation of immune microenvironment after treatment with olaparib alone or in combination with cisplatin or durvalumab in patients with operable head and neck squamous cell carcinoma (HNSCC).

**Experimental Design:**

Forty-one patients with HNSCC were randomized to cisplatin plus olaparib (arm A), olaparib alone (arm B), no treatment (arm C) or durvalumab plus olaparib (arm D). The primary endpoint was to evaluate the percentage of patients in each arm that achieved a reduction of at least 25% in Ki67. Secondary endpoints included objective response rate (ORR), safety, and pathologic complete response (pCR) rate. Paired baseline and resection tumor biopsies and blood samples were evaluated for prespecified biomarkers.

**Results:**

A decrease in Ki67 of at least 25% was observed in 44.8% of treated patients, as measured by quantitative immunofluorescence. The ORR among treated patients was 12.1%. pCR was observed in 2 patients. Two serious adverse events occurred in 2 patients.

Programmed death ligand 1 (PD-L1) levels [combined positive score (CPS)] were significantly higher after treatment in arms A and D. Expression of *CD163* and colony-stimulating factor 1 receptor (*CSF1R*) genes, markers of M2 macrophages, increased significantly posttreatment whereas the expression of *CD80*, a marker of M1 macrophages, decreased.

**Conclusion:**

Preoperative olaparib with cisplatin or alone or with durvalumab was safe in the preoperative setting and led to decrease in Ki67 of at least 25% in 44.8% of treated patients. Olaparib-based treatment modulates the tumor microenvironment leading to upregulation of PD-L1 and induction of protumor features of macrophages.

**Significance:**

HNSCC is characterized by defective DNA repair pathways and immunosuppressive tumor microenvironment. PARP inhibitors, which promote DNA damage and “reset” the inflammatory tumor microenvironment, can establish an effective antitumor response. This phase II WOO trial in HNSCC demonstrated the immunomodulatory effects of PARP inhibitor–induced DNA damage. In this chemo-naïve population, PARP inhibitor–based treatment, reduced tumor cell proliferation and modulated tumor microenvironment. After olaparib upregulation of PD-L1 and macrophages, suggests that combinatorial treatment might be beneficial.

**Synopsis:**

Our WOO study demonstrates that preoperative olaparib results in a reduction in Ki67, upregulation of PD-L1 CPS, and induction of protumor features of macrophages in HNSCC.

## Introduction

Despite multimodal treatment including surgery, radiotherapy and/or chemotherapy, 40% to 60% of patients with locally advanced head and neck squamous cell carcinoma (HNSCC) will eventually relapse (1). With these classical treatment modalities, the maximal tolerable toxicity is reached, limiting further treatment improvement. One way to ameliorate our therapeutic approach is either to replace chemotherapy with a targeted agent to improve the toxicity profile with the same efficacy, or to add a new agent with non–cross-toxicities to increase treatment efficacy.

PARP inhibitors (PARPi) have been shown to be effective against homologous recombination repair deficient tumors (2). Several studies (3, 4), have shown that patients with HNSCC are characterized by aberrant activity of DNA repair pathways, including base excision repair (BER) and DNA double-strand break repair. These deregulated repair mechanisms are implicated in both the pathogenesis of the disease (5) and the outcome of chemotherapy (6). PARPi exploit this deregulation, particularly in tumors with existing DNA repair defects, by increasing DNA damage.

There is substantial evidence that tumor cells use PARP to repair platinum-induced DNA damage and thus escape apoptosis (7). Therefore, the addition of PARPi to platinum-based chemotherapy seems promising in HNSCC. Although the greatest efficacy of PARPi has been observed in tumors with BRCA1/2 mutations, patients without these mutations can also potentially benefit (8–10). PARPi-induced DNA damage can promote immune priming through different molecular mechanisms and enhance adaptive upregulation of programmed cell death ligand 1 (PD-L1) expression in preclinical models (11), resulting in immunologically relevant mutations (12–14).

DNA damage can also induce an innate immune response activating the STING (stimulator of interferon genes) pathway. Cytosolic DNA that results from DNA damage can activate STING leading to enhanced IFN production (15). In recurrent/metastatic HNSCC, treatment with programmed cell death protein 1 (PD-1) checkpoint has shown overall survival benefit in the first- and second-line settings (16).The available preclinical, translational, and clinical trial data strongly support the combination of PARPi with immune checkpoint inhibitors (11, 17–26) in patients with HNSCC.

Window of opportunity (WOO) studies aim to identify new relevant molecular therapeutic targets in HNSCC and facilitate translational research. In OPHELIA, a WOO phase II study, patients were randomized 3:3:1:3 to cisplatin (C) followed by olaparib (O), olaparib alone, no treatment, and durvalumab (D) followed by olaparib. Paired baseline and resection tumor biopsies were evaluated for prespecified biomarkers.

## Materials and Methods

### Study Design and Participants

We conducted a randomized WOO phase II study at three centers, University Hospital “Attikon” (Athens), Hippokrateion University Hospital (Athens), and “THERMI” Clinic (Thessaloniki), in Greece. No formal comparison among the arms was performed. Patients allocated to the olaparib monotherapy arm served as a proof-of-concept to interpret the mechanism of action of olaparib. Patients allocated in the “no treatment” group were used as control. The study was initially designed as a three-arm study and was subsequently amended to include D-Olaparib arm.

Newly diagnosed patients with histologically confirmed squamous cell carcinoma of the oral cavity, oropharynx, hypopharynx, or larynx that were candidates for primary surgical treatment were eligible. Additional eligibility criteria included Eastern Cooperative Oncology Group performance status ≤ 1 and sufficient biological material (tumor tissue and blood) for translational research. The trial was approved by the Institutional Review Boards of the Attikon University Hospital (Athens, Greece), Hippokrateion University Hospital (Athens, Greece), and “THERMI” Clinic (Thessaloniki, Greece) and was completed in accordance with the Protocol and Good Clinical Practice guidelines. Supplementary Table S1A shows the representativeness of study participants. All patients provided written informed consent.

### Randomization and Masking

Patients with operable, histologically documented, squamous cell carcinoma of the oral cavity, oropharynx, hypopharynx, or larynx were randomized 3:3:1:3 among C and olaparib (arm A), monotherapy with O (arm B), no treatment (arm C), or combination with D and olaparib (arm D), before surgery. Patients had surgery on day 23 to day 29. Patients were selected irrespectively of mutation status (somatic and/or germline) or other biomarkers.

### Procedures

The optimal doses of the combination of C with olaparib were determined on the basis of a phase I trial in solid tumors (27). A total of 41 patients were randomized 3:3:1:3 to C 60 mg/m^2^ on day 1 followed by olaparib 75 mg days 1–5 (A), olaparib 300 mg twice a day for 21–28 days (B), no treatment (C), and D 1,500 mg on day 1 followed by olaparib 600 mg daily for 21–28 days (D). Patients had been staged by imaging exams during the last 30 days before enrollment and reassessed by imaging (tumor objective response by RECIST) on the 22nd to 28th day before surgery or second biopsy.

After surgery, all patients were reevaluated by a multidisciplinary tumor board and if necessary were referred for postoperative radiotherapy according to current clinical guidelines (NCCN, guidelines for Head and Neck Cancers). Patients were followed for 3 months after the date of second biopsy or surgery, or after the last dose of study treatment, or the initiation of the next antineoplastic treatment if that occurred earlier. Adverse events were graded according to the NCI Common Terminology Criteria for Adverse Events V. 4.0 scale.

The details for the assays and image analysis are summarized in the Supplementary Materials and Methods.

### IHC

Chromogenic staining was performed (Liquid DAB + Substrate Chromogen, Dako), followed by counterstain with Tacha's hematoxylin (Biocare Medical) for Ki67, PD-L1, STING, γH2AX at Second Department of Pathology, University of Athens Medical School (Attikon University Hospital, Athens, Greece).

### Quantitative Immunofluorescence

We performed two multiplexed quantitative immunofluorescence (QIF) staining protocols for simultaneous detection of (i) cytokeratin (CK)+ tumor cells, non-tumor cells, PD-L1+ cells, STING + cells, and Ki67+ cells and (ii) CK+ tumor cells, non-tumor cells, CD163^+^ and CSF1R+ cells at Rimm Lab, Yale University, Department of Pathology. QIF was performed using the automated quantitative analysis (AQUA) method as described previously (28, 29). AQUA scores, a quantitative score of immunofluorescence intensity for the tumor, were generated and the absolute expression of the markers was determined.

### Quantification of Tumor-infiltrating Lymphocytes

The Aperio ScanScope XT platform was used at × 40 to digitize the slides. QuPath (open source software) was used for cell segmentation based on detection of individual nuclei or cells (30). All tissue sections were visually assessed and cases with staining artifacts or misassignments were omitted from the analysis.

### NanoString nCounter Gene Expression Assay

The nCounter PanCancer IO 360 Panel containing 770 genes related to the tumor, its microenvironment and the antitumor immune response was used (Supplementary Table S1B). A total of 56 out of 59 pretreatment and posttreatment samples (arm A, B, and D) passed all quality control runs and were analyzed.

### Circulating Tumor Cells

For circulating tumor cell (CTC) analysis the size-dependent microfluidic device Parsortix (ANGLE plc) was used and mRNA was isolated using TRIzol-LS (Thermo Fisher Scientific), followed by cDNA synthesis (Applied Biosystems; ref. 31). *PD-L1* transcripts were quantified by qRT-PCR in the Cobas v480 System (Roche) and normalized in respect to beta-2-microglobulin expression in the same cDNAs, using the 2^−ΔΔCt^ approach, as described previously (31, 32).

### Tumor mutational Burden and Next-generation Sequencing Analysis

Genomic analysis of germline and tumor DNA assessed loss-of-function variants in a number of DNA damage response (DDR) pathways (homologous recombination/non-homologous end-joining, nucleotide excision repair, mismatch repair, and BER) genes by next-generation sequencing (NGS). Nonsynonymous somatic tumor mutational burden (TMB) was measured. High TMB was defined as > 100 mut/Mb in OncoDNA assay. OncoDNA sequencing panel was used for NGS analysis of 311 genes commonly involved in HNSCC including homologous recombination deoxyribonucleic acid repair (HRD) genes. Genomic data were obtained from OncoDNA (http://www.oncodna.com), a capture-based sequencing panel of 310 cancer-associated genes.

### Outcomes

The primary endpoint of the study was to evaluate the percentage of patients in each arm that achieved a reduction of at least 25% in Ki67 proliferation index, calculated as the difference of the measured Ki67 in tumor before and after treatment (ΔKi67). ΔKi67 ≥ 25% in pretreatment and posttreatment as a primary endpoint to test the hypothesis that olaparib would potentiate antitumor effects, was based on a previous WOO HNSCC study (33). WOO trials can detect the pharmacodynamic effect of an agent within a tumor. Correlation of this potential surrogate histologic measure (ΔKi67) with physical examination (volumetric) (34), pathology (pathologic downstaging or pathologic complete response; pCR) or imaging is also reported here.

Secondary efficacy endpoints included objective response rate (ORR) according to RECIST 1.1 criteria, and pCR rate. Surgical complication rate and number of participants with tolerability to treatment were the safety endpoints.

### Statistical Analysis

According to the Fleming single-stage design, assuming that the expected percentage of patients with ΔKi67 ≥25% would be at least 40% for each treatment group and the minimum acceptable rate 10%, a total of 12 patients per treatment group provided 90% power with a two-sided α of 10%. Differences among the three treatment groups, pretreatment and posttreatment were evaluated using the Kruskal–Wallis test. If the result of the Kruskal–Wallis was found to be significant, *post hoc* analyses were performed. The levels of the parameters of interest were compared before and after treatment by the Wilcoxon signed-rank test. The Wilcoxon rank-sum test was used to assess differences in biomarkers of interest between responders and non-responder patients. The association of Κi67, STING, PD-L1, and γ-H2ΑΧ values obtained from IHC with the corresponding QIF values was evaluated with Spearman correlations. Differential gene expression analysis per gene or per signature was performed by NanoString using linear models. A mixed linear model for analyses with subject as a blocking factor to account for the temporal effects in the model was used for paired samples analysis. Safety assessment was performed in the safety population consisting of all eligible patients who received at least one dose of the study drug(s).

All tests were two sided, and significance was set at the 5% level of significance. *P* values were adjusted for multiple comparisons using the Benjamini and Hochberg method. Analysis was performed using the SAS version 9.3 and the R studio version 3.5.0.

### Data Availability Statement

Data are available upon reasonable request.

## Results

**FIGURE 1:**
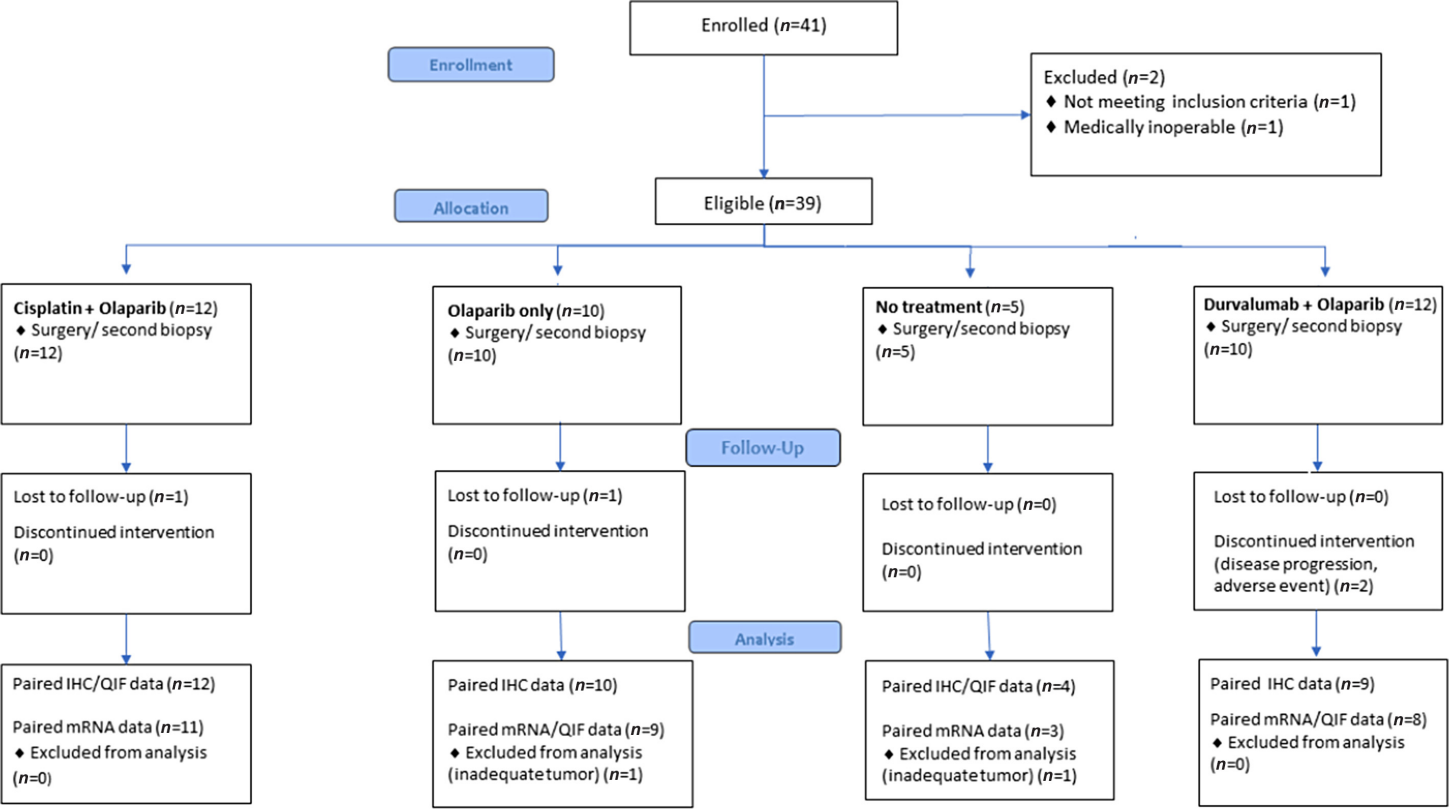
Consort diagram.

Between October 20, 2016 and October 10, 2019, 41 patients were randomly assigned to either C plus olaparib (*n* = 12), olaparib alone (*n* = 12), no treatment (*n* = 5), or D plus olaparib (*n* = 12). Two patients were deemed ineligible and were excluded from the final analysis (Fig. 1). Baseline demographic and relevant clinical variables of the intention-to-treat population were balanced among the treatment arms (Table 1).

**TABLE 1:** Patient and tumor characteristics at baseline

	Cisplatin & olaparib (*N* = 12)	Olaparib only (*N* = 10)	No treatment (*N* = 5)	Olaparib & durvalumab (*N* = 12)	Total (*N* = 39)
Age at study entry					
Median (min, max)	64.7 (51.7, 70.5)	59.8 (47.8, 84.6)	56.3 (54, 67)	68.7 (48.6, 85.9)	61.5 (47.8, 85.9)
	***N* (%)**	***N* (%)**	***N* (%)**	***N* (%)**	***N* (%)**
Sex					
Men	11 (91.7)	7 (70.0)	4 (80.0)	9 (75.0)	31 (79.5)
Women	1 (8.3)	3 (30.0)	1 (20.0)	3 (25.0)	8 (20.5)
PS					
0	11 (91.7)	9 (90.0)	4 (80.0)	11 (91.7)	35 (89.7)
1	1 (8.3)	1 (10.0)	1 (20.0)	1 (8.3)	4 (10.3)
Smoking status					
Non-smoker	0 (0.0)	2 (20.0)	0 (0.0)	2 (16.7)	4 (10.3)
Ex-smoker	9 (75.0)	3 (30.0)	2 (40.0)	6 (50.0)	20 (51.3)
Current smoker	3 (25.0)	5 (50.0)	3 (60.0)	4 (33.3)	15 (38.5)
Alcohol consumption					
Uncommon	3 (25.0)	2 (20.0)	1 (20.0)	6 (50.0)	12 (30.8)
Mild	4 (33.3)	2 (20.0)	1 (20.0)	0 (0.0)	7 (17.9)
Moderate	1 (8.3)	2 (20.0)	1 (20.0)	3 (25.0)	7 (17.9)
Heavy	4 (33.3)	4 (40.0)	2 (40.0)	3 (25.0)	13 (33.3)
Primary tumor site					
Larynx	2 (16.7)	1 (10.0)	0 (0.0)	3 (25.0)	6 (15.4)
Oral cavity/Lips	8 (66.7)	9 (90.0)	4 (80.0)	9 (75.0)	30 (76.9)
Oropharynx	2 (16.7)	0 (0.0)	1 (20.0)	0 (0.0)	3 (7.7)
p16^a^					
Positive	2 (17.0)	0 (0.0)	0 (0.0)	0 (0.0)	2 (66.7)
Negative	0 (0.0)	0 (0.0)	1 (20.0)	0 (0.0)	1 (33.3)
Grade					
1	2 (16.7)	2 (20.0)	1 (20.0)	1 (8.3)	6 (15.4)
2	6 (50.0)	4 (40.0)	2 (40.0)	6 (50.0)	18 (46.2)
3	3 (25.0)	3 (30.0)	2 (40.0)	4 (33.3)	12 (30.8)
4	0 (0.0)	0 (0.0)	0 (0.0)	1 (8.3)	1 (2.6)
Unknown	1 (8.3)	1 (10.0)	0 (0.0)	0 (0.0)	2 (5.1)
Tumor (T) stage					
T1	0 (0.0)	2 (20.0)	1 (20.0)	0 (0.0)	3 (7.7)
T2	3 (25.0)	1 (10.0)	1 (20.0)	7 (58.3)	12 (30.8)
T3	3 (25.0)	3 (30.0)	1 (20.0)	1 (8.3)	8 (20.5)
T4a	6 (50.0)	4 (40.0)	2 (40.0)	4 (33.3)	16 (41.0)
Nodal (N) stage					
N0	3 (25.0)	8 (80.0)	0 (0.0)	9 (75.0)	20 (51.3)
N1	3 (25.0)	0 (0.0)	1 (20.0)	0 (0.0)	4 (10.3)
N2a	1 (8.3)	0 (0.0)	0 (0.0)	0 (0.0)	1 (2.6)
N2b	3 (25.0)	1 (10.0)	3 (60.0)	0 (0.0)	7 (17.9)
N2c	2 (16.7)	1 (10.0)	1 (20.0)	2 (16.7)	6 (15.4)
N3	0 (0.0)	0 (0.0)	0 (0.0)	1 (8.3)	1 (2.6)

^a^Assessed only for tumors located in the oropharynx.

Overall, 37 patients (94.9%) underwent a second biopsy/surgery. One patient treated with D-Olaparib refused to undergo surgery, whereas in one additional patient the tumor was assessed as unresectable due to disease progression following treatment with D-Olaparib. Treatment completion was achieved in 31 of 34 treated patients (91.2%) [12 in the C-Olaparib group (100%), 10 in Olaparib only (100%), and 9 in the D-Olaparib treatment group (75%)]. Treatment discontinuation was noted in 2 patients who received D-Olaparib due to disease progression (*n* = 1) and development of thromboembolic adverse event (*n* = 1), whereas one patient treated with D-Olaparib did not present for follow-up.

### Primary Endpoint (reduction of at least 25% in Ki67)

**FIGURE 2:**
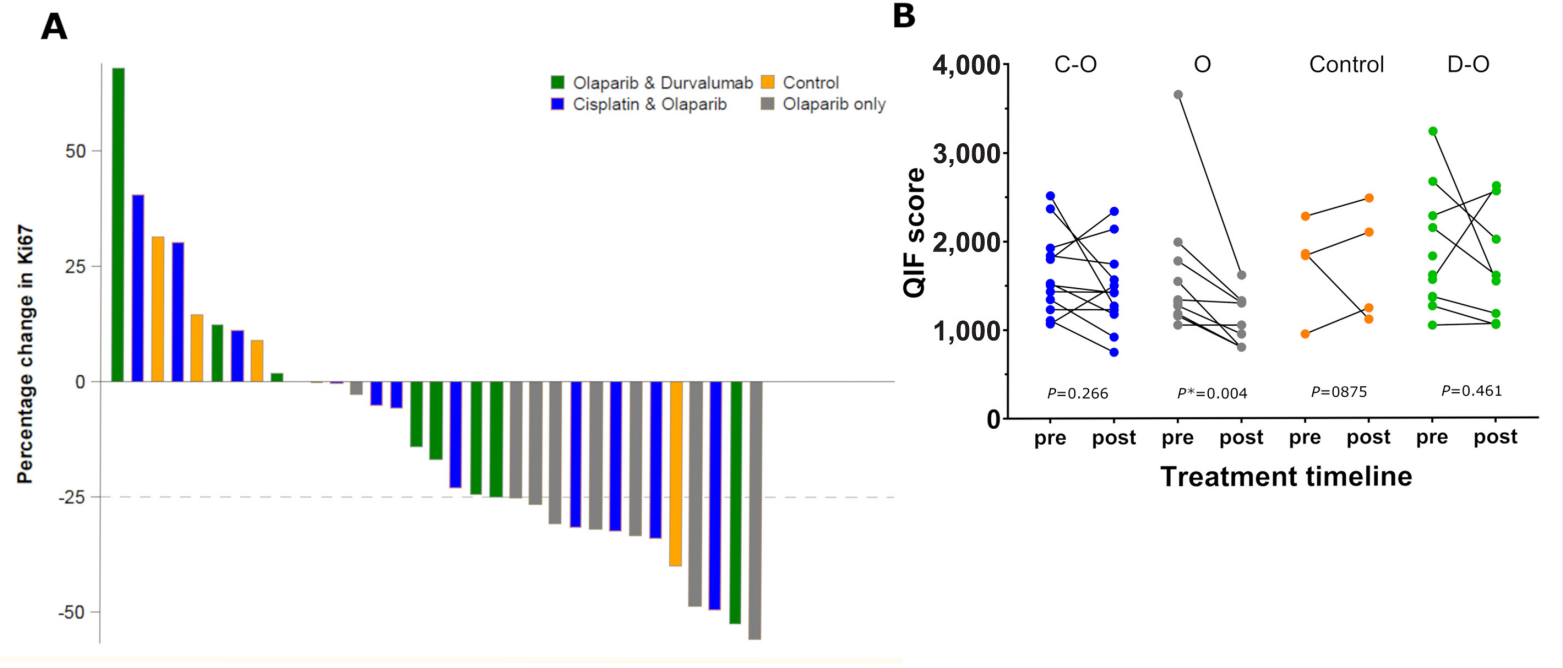
**A,** Waterfall plot of Ki67 change (measured by QIF) compared with pretreatment values. **B,** Comparison of Ki67 distribution assessed by QIF pretreatment and posttreatment/second biopsy or surgery within study groups. Wilcoxon matched pairs signed-rank *P* values were calculated for each treatment group; Cisplatin-Olaparib (C-O), *P* = 0.266; Olaparib (O), *P* = 0.004; Control, *P* = 0.875; Durvalumab-Olaparib (D-O), *P* = 0.461.

Ki67 was decreased after olaparib-based treatment in 23 of 29 (79.3%) available samples when assessed by QIF (35); 13 of 23 had a decrease of at least 25%. A waterfall plot depicting per-patient percent change in Ki67 is shown in Fig. 2A. In the olaparib arm, a significant decrease in Ki67 tumor QIF scores was reported posttreatment (*P* = 0.004; Fig. 2B).

### Secondary Endpoint (response by ORR and pCR)

All ORR was determined by RECIST 1.1. Objective response was achieved in 4 patients (10.3% of the entire cohort); one with complete and 3 with partial responses (PR), while stable disease (SD) was observed in 28 (71.8%) and progressive disease (PD) in 6 patients (15.4%). There were 6 patients who developed disease progression: 3 in the no treatment arm, 1 in C-Olaparib, and 2 in D-Olaparib. Tumor response was not evaluated in 1 patient in the Olaparib only arm who underwent surgery earlier than scheduled and was unable to proceed for reevaluation. The ORR among treated patients evaluable for response (*n* = 33) was 12.1%. The median time to best overall response (as assessed by investigator using the RECIST) was 0.7 months (range, 0.6–1.10) for treated patients with available data (*n* = 32).

pCR was observed in 2 patients; 1 treated with D-Olaparib who also achieved a complete response (CR) and 1 in the C-Olaparib arm. One patient in the Olaparib only arm attained almost pCR (3 mm tumor was detected in surgical biopsy). Thirteen treated patients (38.2%) attained pathologic downstaging.

### Clinical Response

Response based on physical examination, pathology, or imaging was also assessed because traditional RECIST might not be appropriate for short “WOO” biomarker therapeutic trials seeking to identify earlier response biomarkers associated with objective clinical response. In our study, clinical response, defined as response based on physical examination (volumetric tumor measurement; ref. 34) and/or imaging and/or pathology, was observed in 17 patients (43.6% of the entire cohort; 50% of treated patients). Of these 17, 7 had received C-Olaparib, 2 D-Olaparib, and 8 Olaparib only. This analysis was not prespecified in the protocol and was exploratory.

### Exploratory Objectives

#### Tissue Biomarkers

##### Proteomics

**FIGURE 3:**
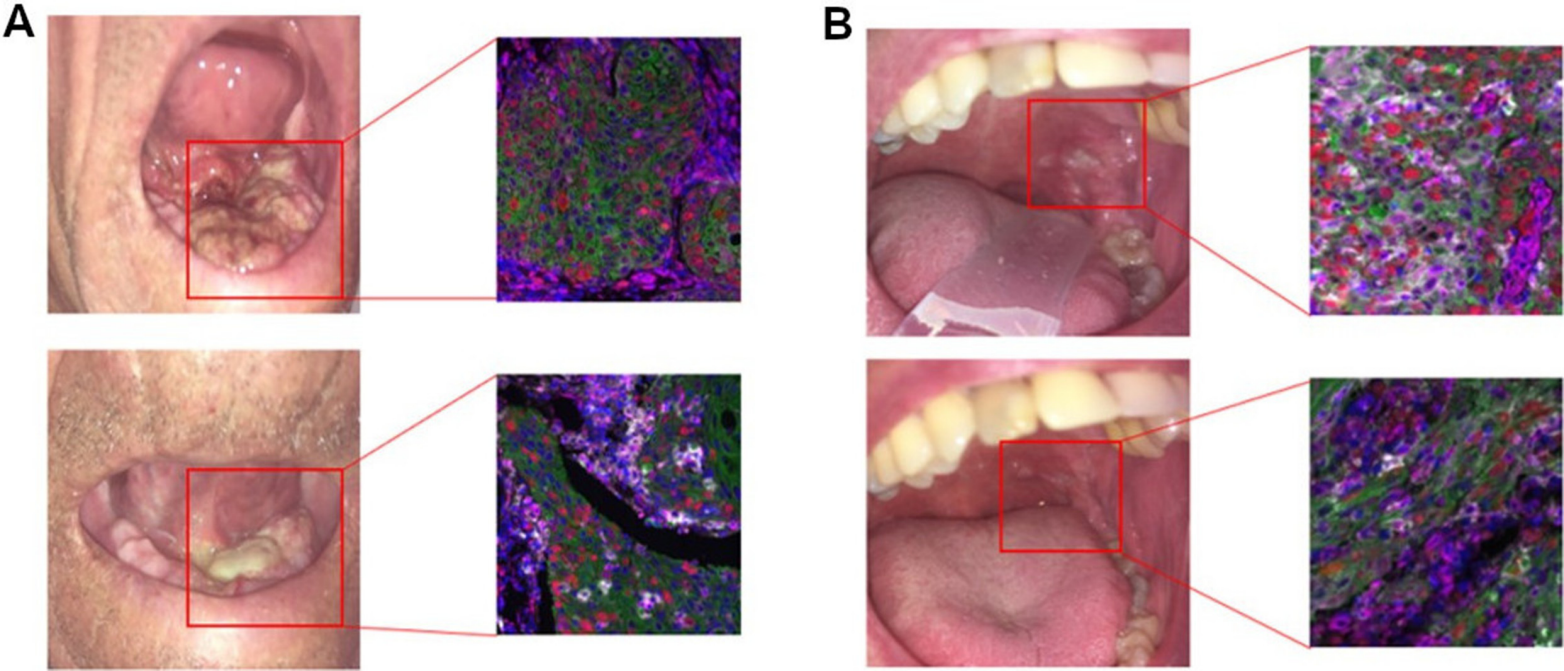
Baseline (up) and after treatment (down) images in Patient A (Cisplatin-Olaparib arm) and B (Olaparib arm), respectively, showing dramatic tumor volumetric decrease. Both patients had SD on imaging based on RECIST. Representative immunofluorescence (IF) images at baseline (up) and after treatment (down), highlighting changes in Ki67 (red), PD-L1 (white), STING (magenta) in tumor. Nuclei (blue) and cytokeratin (green) are also shown. A posttreatment decrease in Ki-67 (measured by QIF) was noticed in both patients.

Tissue PD-L1 expression pre- and post-olaparib–based treatment was assessed by combined positive score (CPS). PD-L1 CPS was higher in 74.2% posttreatment samples in all three olaparib-treated arms, but not in the control arm. CPS was higher in 8 out of 12 tissue samples (*P* = 0.020) in C-Olaparib arm, 7 out of 9 (*P* = 0.039) in D-Olaparib, and 8 out of 10 in Olaparib arm (*P* = 0.17) compared with baseline. No significant differences were observed in terms of STING and γ-H2AX levels before or after treatment. Figure 3 shows the images of cancerous lesions at baseline and after treatment, highlighting concurrent changes in Ki67, PD-L1, and STING in tumor.

##### Transcriptomics

**FIGURE 4:**
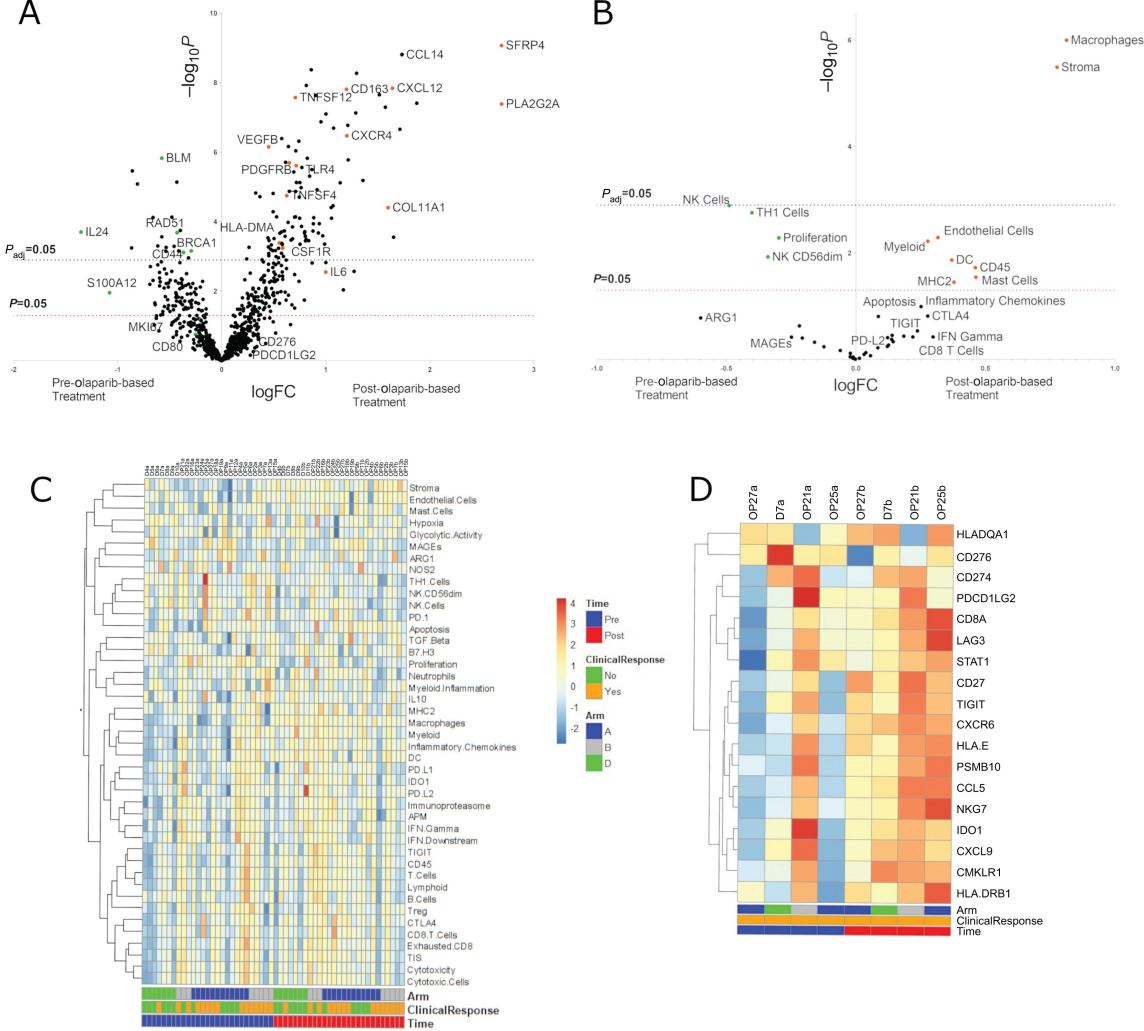
Differentially expressed genes (**A**) and gene signatures (**B**) in pre-olaparib–based treatment relative to post-olaparib–based treatment samples. The significance (*P* value, *P* and adjusted *P* value, *P*_adj_) is represented relative to fold change (FC) in the *x*-axis. Annotated markers are highlighted with orange (increased) or green (decreased). **C,** “All Signatures” heat map shows relatedness among signature scores for each sample. **D,** Subgroup analysis looking at the 4 patients with CR (1), PR (2), and pCR (1) revealed an increase in “Genes Within Tumor Inflammation Signature (TIS)” score posttreatement.

Changes to gene expression patterns and signature scores at baseline and after treatment can reflect a changing tumor microenvironment (TME) and may be useful for understanding the impact of olaparib on tumor cells. By comparing signature scores after treatment to the scores at baseline, changes in the tumor-immune landscape can be visualized. In this study, we found that macrophage and stromal tissue signatures were significantly higher post-olaparib–based treatment (*P* adjusted, *P*_adj_ < 0.05). Signatures of endothelial cells, myeloid, dendritic cells, and CD45 were increased posttreatment (*P* < 0.05) whereas natural killer (NK) cells, Th type 1 (Th1) cells, and proliferation were decreased posttreatment (*P* < 0.05), in all treatment arms. Looking at the expression of individual genes of the macrophage signature, we noticed that expression of *CD163*, a marker of M2 macrophages increased significantly posttreatment (logFC 1.198*, P*_adj_ < 0.0001) whereas the expression of *CD80*, a marker of M1 macrophages, decreased (logFC −0.2532, *P* = 0.17). We also noticed a posttreatment increase in colony-stimulating factor 1 receptor (*CSF1R*), which is usually expressed on M2 macrophages (logFC 0.58, *P*_adj_ = 0.029). Genes involved in antigen presentation (*HLA-DMA*), chemokine- and cytokine-signaling cascades (*CCL14,CXCL12, CXCR4*)*,* and Toll-like receptors (*TLR4*) were also upregulated, suggesting aberrant myeloid activity. Costimulatory molecule *TNFSF4* (OX40L) was significantly upregulated posttreatment (*P*_adj_ < 0.05). In addition, olaparib-based treatment resulted in a significant increase in genes involved in angiogenesis (*PDGFRB, VEGFB, COL11A1*; *P*_adj_ < 0.05). *CD274* (PD-L1) and *PDCD1LG2* (PD-L2) expression did not differ significantly pretreatment and posttreatment (logFC −0.17, *P* = 0.42, and logFC 0.10, *P* = 0.59, respectively). Increases in transcripts among genes related to proinflammatory cytokine signaling (*IL32*), lymphocytes (*CD4*) as well as those encoding for apoptosis (*BCL2*) were also observed posttreatment (*P*_adj_ < 0.05; Fig. 4; Supplementary Fig. S1; Supplementary Tables S2 and S3). Because we were underpowered to identify biomarkers of response based on ORR (CR = 1, PR = 2, SD = 25, PD = 1), we sought to identify differential gene expression between patients who had a response based on physical exam and/or imaging and/or pathology (*N* = 27) and those who did not (*N* = 14). Pretreatment samples of responders had higher inflammatory chemokine (AUC = 0.74) and exhausted CD8 (AUC = 0.69) signature scores than non-responders (*P* < 0.05). Cytotoxic cells, PD-1, CD45, CD8 T cells, Lymphoid, CTLA4, IFN gamma signature scores were also higher in responders compared with non-responders, but this comparison did not reach statistical significance (*P* < 0.1; Supplementary Fig. S2; Supplementary Tables S4A and S4B).

### Tumor-infiltrating lymphocytes

The presence of tumor-infiltrating lymphocytes (TIL) is increasingly recognized as an important biomarker in HNSCC (36). Using the QuPath open-source software we were able to measure stromal TILs, calculated as the proportion of TILs over stromal cells; esTILs % = (TILs/total cells – tumor cells) * 100. No significant differences in stromal TILs were found between pretreatment and posttreatment samples in all arms (Supplementary Fig. S3). An increase in esTILs in 5 out of 7 evaluable pairs was observed in the D-Olaparib arm posttreatment but was not statistically significant (*P* = 0.37).

### CTCs Evaluated for PD-L1

PD-L1 in CTCs was assessed in 9 out of 12 patients in the D-Olaparib arm (26.5% of all treated patients) and 5 of them were PD-L1 negative (55.6%) before treatment. After D-Olaparib treatment, 8 out of 9 patients were PD-L1 negative (88.9%) and 1 patient became PD-L1 positive. No association was detected between PD-L1 expression in CTCs and PD-L1 levels as measured by IHC (*P* = 0.43), or QIF in tumor (*P* = 0.89), probably due to the small number of patients with available data (Supplementary Fig. S4).

### Evaluation of the Mutational Profile Pretreatment and Posttreatment

A total of 79 mutations were detected in 30 patients (76.9%) at the time of first biopsy (range, 1–6; median 2). All 12 patients in the C-Olaparib group carried mutations at the time of first biopsy. Likewise, more than half of the patients in the D-Olaparib (7/12 patients; 58.3%) and Olaparib only (8/10 patients; 80%) group had mutations prior to treatment administration with most of them being identified in the *TP53* gene (18 mutations). Mutations in DDR and chromatin remodeling genes were identified in 5 (12.8% of total; 16.7% of mutated) and 8 (20.5% of total; 26.7% of mutated) patients, respectively. Baseline TMB was measured in 17 patients; none of them had high TMB (>100 mut/Mb; Supplementary Fig. S5).

### Association of Response with Mutations and Other Biomarkers

Response based on physical exam, imaging, or pathology was noted in 3 of 4 treated patients with mutations in DDR genes before therapy and in 6 of 7 patients with mutations in chromatin remodeling genes. Out of 10 patients with mutations in DDR or chromatin remodeling genes before treatment, 8 achieved a tumor response based on physical exam, imaging or pathology.

No significant association was found between response based on physical exam, imaging or pathology and changes in Ki67, STING, and PD-L1 (ΔKi67, ΔSTING, ΔPD-L1) or Ki67, STING, PD-L1 and γ-H2Ax values, and TMB at baseline.

### Safety Analysis

All eligible patients who received at least one dose of the study treatment were assessed for safety (*N* = 34). Two serious adverse events occurred in 2 patients (5.9%): one grade 3 postoperative hemorrhage in a patient treated with C-Olaparib and one grade 3 thromboembolic event in a patient treated with D-Olaparib that was assessed as a suspected unexpected serious adverse reaction, related to treatment (Supplementary Table S5).

## Discussion

To the best of our knowledge, we report the first trial that evaluates the efficacy of olaparib alone or with C or with D in the preoperative setting in patients with operable HNSCC. Our data indicate that olaparib-based treatment enhances both antitumor and protumor features. Olaparib-induced DNA damage leads to decrease in proliferation (measured by Ki67), enhanced immune priming and induced adaptive upregulation of PD-L1 expression. This modulation in HNSCC TME results in increased levels of intratumoral macrophages (*CD163, CSF1R*) that might act as a liability of PARPi treatment.

The primary endpoint of the study was to evaluate the percentage of patients in each arm that achieved a reduction of at least 25% in Ki67 posttreatment. Thirteen of 29 (45%) had a decrease of at least 25% whereas a decrease in proliferation was observed in the majority of treated samples (23/29, 79%). However, ΔKi67 was not associated with response (based on physical exam, imaging, or pathology). ΔKi67 may not be the preferred surrogate marker in HNSCC WOO trials. Change in tumor size rather than ΔKi67 was the preferred surrogate marker in a placebo-controlled window study of EGFR, Src, or combined blockade in operable HNSCC by Bauman and colleagues (37). In the era of immune checkpoint inhibitors, the selection of endpoints for WOO studies in HNSCC becomes even more complicated. Volumetric tumor response is selected by Schoenfeld and colleagues (38) whereas pathologic tumor response is selected by Uppaluri and colleagues (39).

Olaparib appears to demonstrate activity in HNSCC especially in tumors with alterations in genes involved in DNA repair or chromatin remodeling. The patient who attained pCR to olaparib had loss-of-function mutation of the AT-rich interactive domain 2 (*ARID2*) gene, which is involved in chromatin structure and modification. *ARID2* deficiency has been linked to PARPi sensitivity in lung cancer (40). A patient who developed response to olaparib as per physical examination, not meeting the criteria RECIST 1.1, had a *CHEK2* inactivating mutation. A patient who developed PR to C-Olaparib had an inactivating mutation in lysine‐specific methyltransferase 2C (*KMT2C, MLL-3*) gene. Low KMT2C activity is associated with DDR deficiency and PARPi sensitivity (41).

Defects in DDR pathways result in accumulation of cytosolic DNA, triggering activation of STING and promoting type I immunity (15). Moreover, DNA damage can lead to adaptive upregulation of PD-L1 expression (11). To understand the distinct effects that PARPi-induced DNA damage may have on tumor immunogenicity we combined genomic profiling with gene expression profiling and IHC/QIF assessments of DNA repair- and immune-related biomarkers. Our goal was to study the overlap between DDR and immune-related biomarker clusters and to develop a better understanding of how DNA damage interfaces with antitumor immunity.

PD-L1 expression (by CPS) increased after treatment in all three treated groups (C-Olaparib, Olaparib, and D-Olaparib) and was significantly higher in C-Olaparib (*P* = 0.020) and D-Olaparib (*P* = 0.039). No differences were observed in terms of STING levels among or within treatment groups before and after treatment. There was a significant posttreatment increase in the myeloid activity in all treated arms. *CD163* a marker of immune-suppressive M2-like macrophages (protumor phenotype) and *CSF1R*, which promotes macrophage infiltration (42), were significantly upregulated after olaparib-based treatment (Supplementary Table S3; Supplementary Fig. S6). These results are consistent with data showing that macrophage-mediated immunosuppression is a consequence of PARPi treatment in triple-negative breast cancer; targeting immunosuppressive macrophages overcomes PARPi resistance in BRCA1-associated triple-negative breast cancer (43). Furthermore, simultaneous CSF1R targeting might increase the response rate to immune checkpoint blockade (44).

Through multi-omics profiling, we showed that olaparib-based treatment enhances both antitumor and protumor features and modulates the TME in HNSCC. An ongoing study will evaluate the efficacy of PARP/PD-L1 blockade (NCT04169841) and explore whether immunotherapy increases the duration of response to PARP inhibition in HNSCC or whether a third blocking agent such as a CSF1R inhibitor might be necessary to enhance innate and adaptive antitumor immunity and survival in patients with HNSCC.

To conclude, at the current WOO study we found that preoperative olaparib-based treatment was safe in HNSCC and led to a decrease of Ki-67 of at least 25% in a substantial proportion of patients. Our biomarker results demonstrate that olaparib upregulates PD-L1 and M2 macrophages. Given that pembrolizumab is approved in first-line recurrent/metastatic setting either as monotherapy in those expressing CPS ≥ 1 or combined with chemotherapy, olaparib-PD-1 inhibitor combinations deserve further study. The immune stimulating effects of olaparib are antagonized by the upregulation of M2 macrophages and combinatorial approaches including macrophage/CSF1R-targeting therapies, anti-PD-L1, and olaparib may prove effective.

## Supplementary Material

Supplementary Materials and Methods The details for the assays and image analysis are summarized in the Supplementary Materials and Methods.

Supplementary Table 1 Supplementary Table 1a. Representativeness of Study Participants Supplementary
Table 1b. Nanostring IO360 panel

Supplementary Table 2 Supplementary Table 2. Signature scores with significanlty changes pre- and post-Olaparib based treatment

Supplementary Table 3 Supplementary Table 3. Genes with significanlty changes pre- and post-Olaparib based treatment

Supplementary Table 4 Table 4a. Fold-change values for each signature between Response and No Response based on physical examination, pathology or imaging
Table 4b. Fold-change values for significantly differentially expressed genes between Response and No Response based on physical examination, pathology or imaging

Supplementary Table 5 Supplementary Table 5. Incidence of adverse events by maximum grade in the safety population.

Supplementary Figure 1 Supplementary Figure 1. Differentially expressed genes (Left) and gene signatures (Right) Cisplatin-Olaparib (A, B), Olaparib (C,D) and Durvalumab-Olaparib (E, F) arms in pre- and post-treatment samples. The significance (p-value, P and adjusted p-value, Padj) is represented relative to Fold Change (FC) in the x-axis.

Supplementary Figure 2 Supplementary Figure 2. Differentially expressed signatures in Responders relative to no-Responders. Response is based on physical examination, pathology or imaging. Responders had higher scores in Inflammatory Chemokines and Exhausted CD8 Signatures, as well as PD-1, Cytotoxicity and CD45. The significance (p-value, P and adjusted p-value, Padj) is represented relative to Fold Change (FC) in the x-axis.

Supplementary Figure 3 Supplementary Figure 3. A. No significant differences in stromal TILs were found between pre- and post-treatment samples in all arms. In Arm D TILs were increased post-treatment in most of the samples (5 out of 7). Figures B and C show representative images of TILs in pre- and post-treatment samples respectively from one patient in Arm D, measured by QuPath v0.3.0. Figures show tissues before (left) and after (right) QuPath annotations. Color legend; red, tumor cells; purple, lymphocytes; green, fibroblasts; yellow, other; Cisplatin-Olaparib, C-O; Olaparib, O; Durvalumab-Olaparib, D-O

Supplementary Figure 4 Supplementary Figure 4. Relative fold change (2-ΔΔCq) of PD-L1 in respect to Β2Μ (reference gene) in the CTC fraction for individual samples of HNSCC patients before (blue) and after (orange) therapy.

Supplementary Figure 5 Supplementary Figure 5. Map showing the distribution of mutations per gene per tumor. Light and dark purple, green and orange indicate pre- and post-treatment samples for patients treated with cisplatin and olaparib, olaparib and durvalumab and olaparib only, respectively. Blues correspond to samples before and after second biopsy/surgery for patients who did not receive treatment.

Supplementary Figure 6 Supplementary Figure 6. Posttreatment CD163 (by QIF) increase in three patients with concurrent increase in the CD163 transcripts. Representative images from a patient’s tumor tissue sample showing pre and post treatment (Durvalumab-Olaparib Arm) CD163 expression. Nuclei (blue), Cytokeratin (green), CD163 (yellow) and CSF1R (red).
