# Supplementary material for: Phase II Window Study of Olaparib Alone or with Cisplatin or Durvalumab in Operable Head and Neck Cancer
Source: Cancer Res Commun. 2023 Aug 10;3(8):1514–23. doi: 10.1158/2767-9764.CRC-23-0051 (PMC10414130; doi:10.1158/2767-9764.CRC-23-0051)
Supplement: Supplementary Materials and Methods — The details for the assays and image analysis are summarized in the Supplementary Materials and Methods. [file crc-23-0051-s01.docx]

**Supplementary Materials and Methods**

**Omics Analysis**

**Immunohistochemistry (IHC) analysis**

Chromogenic staining was performed (Liquid DAB + Substrate Chromogen, Dako), followed by counterstain with Tacha’s hematoxylin (Biocare Medical, Concord, CA, USA) for three minutes and dehydration with ethanol and xylene**.** Antigen retrieval (EnVision Flex) detection was used either with a citrate solution (pH6.0) or EDTA (pH8.0) on the Dako Autostainer, according to manufacturer’s manual, at 2^nd^ Department of Pathology, University of Athens Medical School (Attikon University Hospital).

Expression of Ki67, PD-L1, STING and γH2AX was evaluated using monoclonal antibodies. Isotype-specific primary antibodies were used to detect Ki67 + cells (recombinant monoclonal mouse antibody, clone MIB-1, Dako, Inc, 1:100), PD-L1+ cells (22C3 antibody concentrate ref: M365329; Dako, Inc, 1:50), STING (recombinant monoclonal antibody Anti-TMEM173 [SP338] ΑΒ227704, abcam, 1:100), γH2AX (monoclonal rabbit antibody, Ser139 #9718, Cell Signalling, 1:100).

For the assessment of Ki67 the percentage of positive tumor cells was calculated focusing on ‘hot spots’. Combined Positive Score (CPS) [(PD-L1 positive tumour cells, lymphocytes, macrophages/ total number of viable tumor cells) x100] was measured both by continuous values and using clinically relevant cutpoints to establish 3 distinct groups (CPS<1, CPS=1-19, CPS≥20). STING was evaluated with a scoring system (ranging from 0 to 12) based on the percentage of positive tumor cells [<5% (0), 5-25% (1), 26-50% (2), 51-75% (3), >75% (4)] multiplied by the staining intensity [no staining (0), weak (1), moderate (2), strong (3)]. For the γH2AX assay, H score was based on the percentage of cells with specific staining intensity [absent (0), weak (1), moderate (2) and strong (3)], [H-score = 0 x (% of cells at 0) + 1 x (% of cells at 1) + 2 x (% of cells at 2) + 3 x (% of cells at 3)].

**Quantitative Immunofluorescence (QIF) analysis**

Formalin-Fixed Paraffin-Embedded whole tissue sections were deparaffinized with xylene. Rehydration was performed using ethanol. Antigen retrieval was performed using EDTA buffer pH 8.0 (for PD-L1, STING, Ki67) and Citrate buffer pH 6 (for CD163, CSF1R) at 97°C for 20 minutes in a pressure-boiling container (PT Module, Lab Vision, Thermo Scientific, Waltham, MA) at RimmLab, Pathology Department, Yale University. 2.5% hydroxyl peroxide in methanol for 30 minutes was used to block endogenous peroxidase activity, followed by 0.3% bovine serum albumin in 0.1mol/L of Tris-buffered saline for 30 minutes at room temperature. This was followed by incubation of the slides with the primary antibodies and cytokeratin. Slides were then incubated at 1-hour room temperature with the primary antibodies and with cytokeratin. Secondary antibodies and tyramide-bound fluorophores were used for target antibody detection. 4,6-diamidino-2-phenyl-indole (DAPI) was used to stain nuclei and mounted with Prolong Gold antifade mounting reagent (P36394, Life Technologies). All staining was performed using the Lab Vision Autostainer 720 (Thermo Scientific).

Fluorescence Measurement and Scoring

Quantitative immunofluorescence (QIF) was assessed using the PM2000 (HistoRx) automated fluorescence microscopy platform. The final images were analyzed and quantified using the AQUAnalysis Software (Navigate Biopharma).

Image analysis with AQUA method of QIF was used for two panels ; Panel A consisted of three markers (PD-L1, STING and Ki67) and Panel B for 2 (CD163, CSF1R). For PD-L1 expression evaluation, E1LN3 (Cell Signaling Technology, Danvers, MA, 873.9 ug/mL), STING, OTI4H1 (ThermoFisher, Scientific, Waltham, MA USA, 2ug/ml), Ki67, MIB-1 (DAKO, Carpinteria, CA, 0.46ug/ml), CD163, clone CD163-L-U (Leica, Novocastra, Wetzlar, Germany, 0.006 μg/mL) and CSF1R, CSF1 receptor (Cell Signaling Technology, Rabbit IgG, clone E4T8Z, 1:1000). A QIF score was generated by dividing the sum of target pixel intensities by the area of the molecularly designated compartment, as previously described ^1^. In order to distinguish tumor from tissue stroma and other components, an epithelial tumor “mask” was created by binarizing the cytokeratin signal and creating an epithelial compartment. Stromal “mask” is created by subtracting tumor mask from the nuclear mask and is representative of tumor microenvironment. The expression level of a maximum of 3 targets can be measured in the above molecular compartments.

For the whole sections, using a × 20 objective, a series of image fields of view (FOV) were captured within the circled invasive tumor to ultimately cover the tissue of interest. Depending on the size of the tumor, 40–200 fields were captured per section. QIF scores were normalized to the exposure time and bit depth at which the images were captured, to compensate for any variability. All acquired histospots were visually assessed and cases with staining artifacts or less than 2% tumor (cytokeratin staining) were omitted from the analysis. Cell nuclei were visualized by the signal from DAPI stain; cytokeratin was visualized with Alexa 546 fluorophore; and the proteins of interest were visualized with the Cy5 (STING, CSF1R), Cy3+ (CD163), Cy7 (PD-L1) and FITC (Ki67) dye.

Validation of staining and protein expression

Yale-Tissue microarray (YTMA) 419, a cell-line tissue microarray with lymph node tissue, head and neck cancer tissue and FaDU cell lines, was used as positive control and for day-to-day standardization of assays. It revealed the expected levels and expression patterns of the biomarkers of interest. QIF scores for each marker, showed high concordance and remained reproducible, among control slides stained in different batches indicating limited inter-batch variation.

**Hematoxylin & Eosin (H & E) staining**

To count total tumor-infiltrating lymphocytes (TILs) H & E staining was performed. Whole tissue serial sections were stained with Hematoxylin (Dako) for 5 minutes, followed by Eosin Y for 60 seconds. The brightfield H & E images were digitized at 20X using the ScanScope AT2 platform (Leica Biosystems, Wetzlar, Germany) at RimmLab, Yale.

Assessment of automated TILs in stroma

QuPath open-source software (version 0.3.0) was used to measure TILs in stroma. We refined the H&E stain estimates for each digitized slide using the “estimate stain vectors” function in QuPath after uploading H&E images. This step is required to normalize for staining variations between images. This is followed by cell segmentation using standardized watershed cell detection parameters ^2^. Next, we trained QuPath to identify tumor cells, immune cells, fibroblasts and other cells (false detections and background) by selecting training objects from each specimen. Classifiers were individually trained for each biopsy and applied on the same biopsy only. We selected areas of interest by excluding those with tissue artifacts e.g., necrosis. Finally, cell classification was quality controlled for optimal performance. The machine-defined stromal TILs variable (esTILs%) was calculated by measuring the proportion of TILs over stromal cells: esTILs %= (TILs/total cells – tumor cells) * 100. This method mimics the manual pathologist scoring of stromal TILs according to the International Immuno-Oncology Biomarker Working Group on Breast Cancer ^3^ and offers an objective quantification of the TILs population rather than an eye estimation.

**NanoString nCounter Gene Expression Assay**

Total RNA was isolated from 2 serial sections of 5-μm-thick FFPE sections of 59 tumors fixed on positively charged slides using the High Pure FFPET RNA Isolation Kit (Roche) following the manufacturer’s protocols. RNA was quantified using the NanoDrop ND1000 spectrophotometer (Thermo Fisher Scientific) at RimmLab, Yale. The nCounter PanCancer IO 360™ Panel (NanoString Technologies) containing 770 genes related to the tumor, its microenvironment and the antitumor immune response was used. (Supplementary Table 1). Per sample, 250 ng of total RNA in a final volume of 5 μl was mixed with a 3′ biotinylated capture probe and a 5′ reporter probe and tagged with a fluorescent barcode from the custom gene expression code set. Probes and target transcripts were hybridized at 67°C for 16–24 hours per manufacturer’s recommendations. Hybridized samples were run on the NanoString nCounter preparation station using the high-sensitivity protocol, in which excess capture and reporter probes were removed and transcript-specific ternary complexes were immobilized on a streptavidin-coated cartridge. The cartridge was scanned at maximum scan resolution on the nCounter Digital Analyzer. Gene expression was normalized to 20 housekeeping genes. Genes with raw counts below the negative controls were removed from the analysis. Genes were normalized using a ratio of the expression value to the geometric mean of all housekeeping genes on the panel. Genes in the Tumor Inflammation Signature (TIS) were normalized using a ratio of the expression value to the geometric mean of the housekeeper genes used only for the TIS signature. Genes were additionally normalized using a ratio of the housekeeper-normalized data and a panel standard run on the same cartridge as the observed data.

1. Camp RL, Chung GG, Rimm DL: Automated subcellular localization and quantification of protein expression in tissue microarrays. Nat Med 8:1323-7, 2002

2. Malpica N, de Solórzano CO, Vaquero JJ, et al: Applying watershed algorithms to the segmentation of clustered nuclei. Cytometry 28:289-97, 1997

3. Salgado R, Denkert C, Demaria S, et al: The evaluation of tumor-infiltrating lymphocytes (TILs) in breast cancer: recommendations by an International TILs Working Group 2014. Annals of Oncology 26:259-271, 2015
