# Supplementary material for: Phase II Window Study of Olaparib Alone or with Cisplatin or Durvalumab in Operable Head and Neck Cancer
Source: Cancer Res Commun. 2023 Aug 10;3(8):1514–23. doi: 10.1158/2767-9764.CRC-23-0051 (PMC10414130; doi:10.1158/2767-9764.CRC-23-0051)

**Supplementary Figure 1.** Differentially expressed genes (Left) and gene signatures (Right) Cisplatin-Olaparib (A, B), Olaparib (C,D) and Durvalumab-Olaparib (E, F) arms in pre- and post-treatment samples. The significance (p-value, P and adjusted p-value, Padj) is represented relative to Fold Change (FC) in the x-axis.


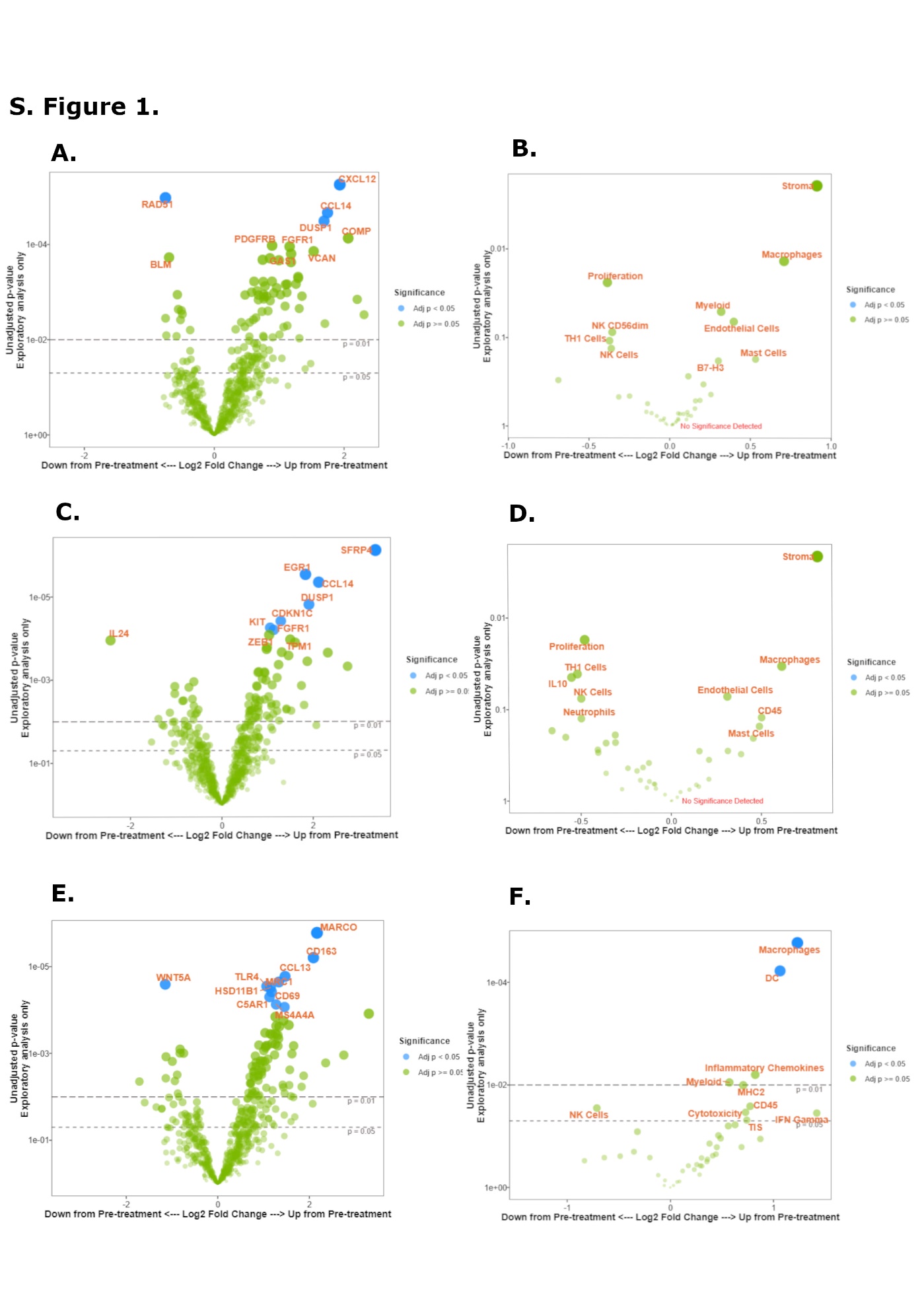

Supplement: Supplementary Figure 1 — Differentially expressed genes (Left) and gene signatures (Right) Cisplatin-Olaparib (A, B), Olaparib (C,D) and Durvalumab-Olaparib (E, F) arms in pre- and post-treatment samples. The significance (p-value, P and adjusted p-value, Padj) is represented relative to Fold Change (FC) in the x-axis. [file crc-23-0051-s07.docx]
