# Supplementary material for: Phase II Window Study of Olaparib Alone or with Cisplatin or Durvalumab in Operable Head and Neck Cancer
Source: Cancer Res Commun. 2023 Aug 10;3(8):1514–23. doi: 10.1158/2767-9764.CRC-23-0051 (PMC10414130; doi:10.1158/2767-9764.CRC-23-0051)

**Supplementary Figure 2**. Differentially expressed signatures in Responders relative to no-Responders. Response is based on physical examination, pathology or imaging. Responders had higher scores in Inflammatory Chemokines and Exhausted CD8 Signatures, as well as PD-1, Cytotoxicity and CD45. The significance (p-value, *P* and adjusted p-value, *Padj*) is represented relative to Fold Change (FC) in the x-axis.


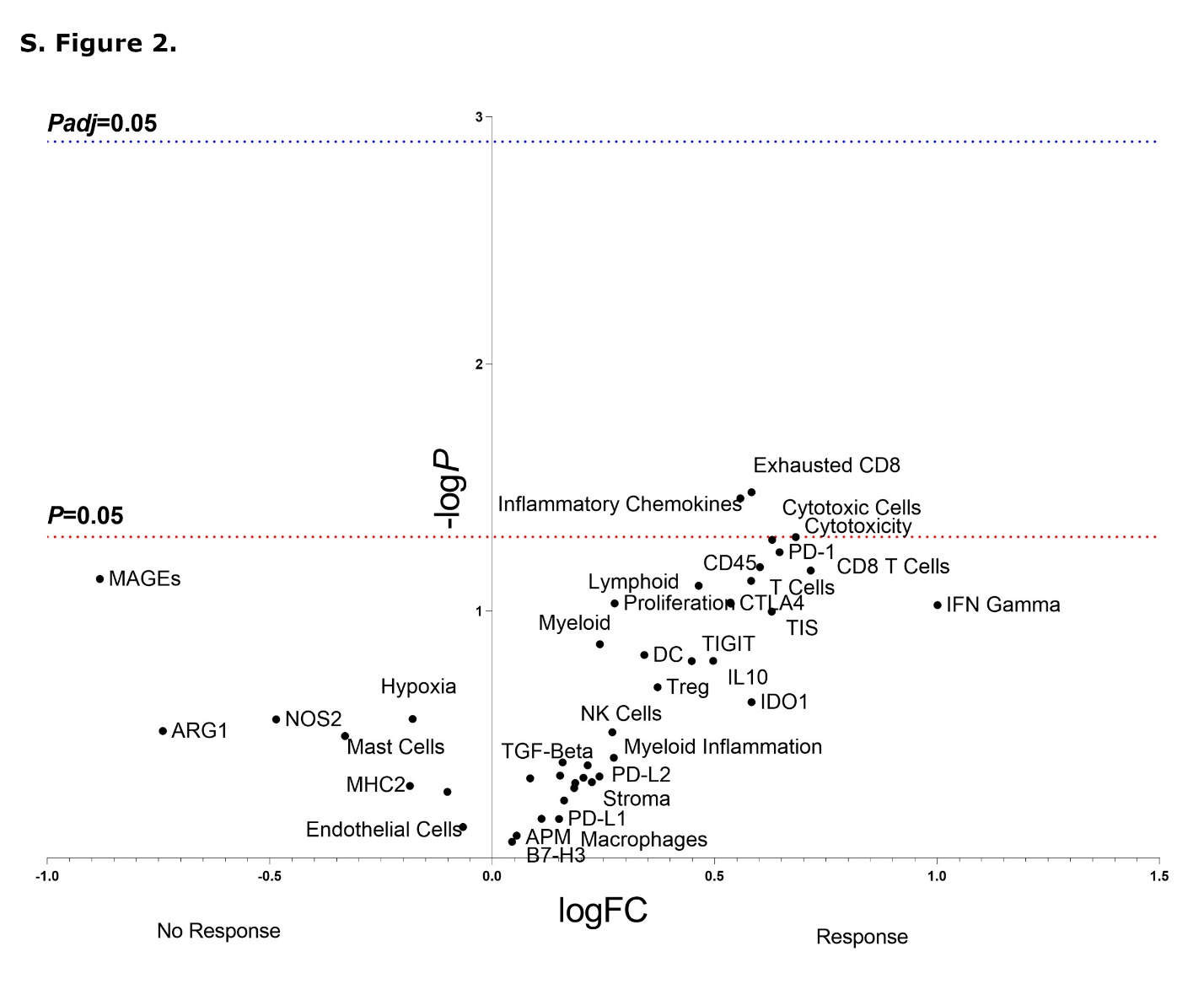

Supplement: Supplementary Figure 2 — Differentially expressed signatures in Responders relative to no-Responders. Response is based on physical examination, pathology or imaging. Responders had higher scores in Inflammatory Chemokines and Exhausted CD8 Signatures, as well as PD-1, Cytotoxicity and CD45. The significance (p-value, P and adjusted p-value, Padj) is represented relative to Fold Change (FC) in the x-axis. [file crc-23-0051-s08.docx]
