# Supplementary material for: Phase II Window Study of Olaparib Alone or with Cisplatin or Durvalumab in Operable Head and Neck Cancer
Source: Cancer Res Commun. 2023 Aug 10;3(8):1514–23. doi: 10.1158/2767-9764.CRC-23-0051 (PMC10414130; doi:10.1158/2767-9764.CRC-23-0051)

**Supplementary Figure 3.** A. No significant differences in stromal TILs were found between pre- and post-treatment samples in all arms. In Arm D TILs were increased post-treatment in most of the samples (5 out of 7). Figures B and C show representative images of TILs in pre- and post-treatment samples respectively from one patient in Arm D, measured by QuPath v0.3.0. Figures show tissues before (left) and after (right) QuPath annotations. Color legend; red, tumor cells; purple, lymphocytes; green, fibroblasts; yellow, other; Cisplatin-Olaparib, C-O; Olaparib, O; Durvalumab-Olaparib, D-O


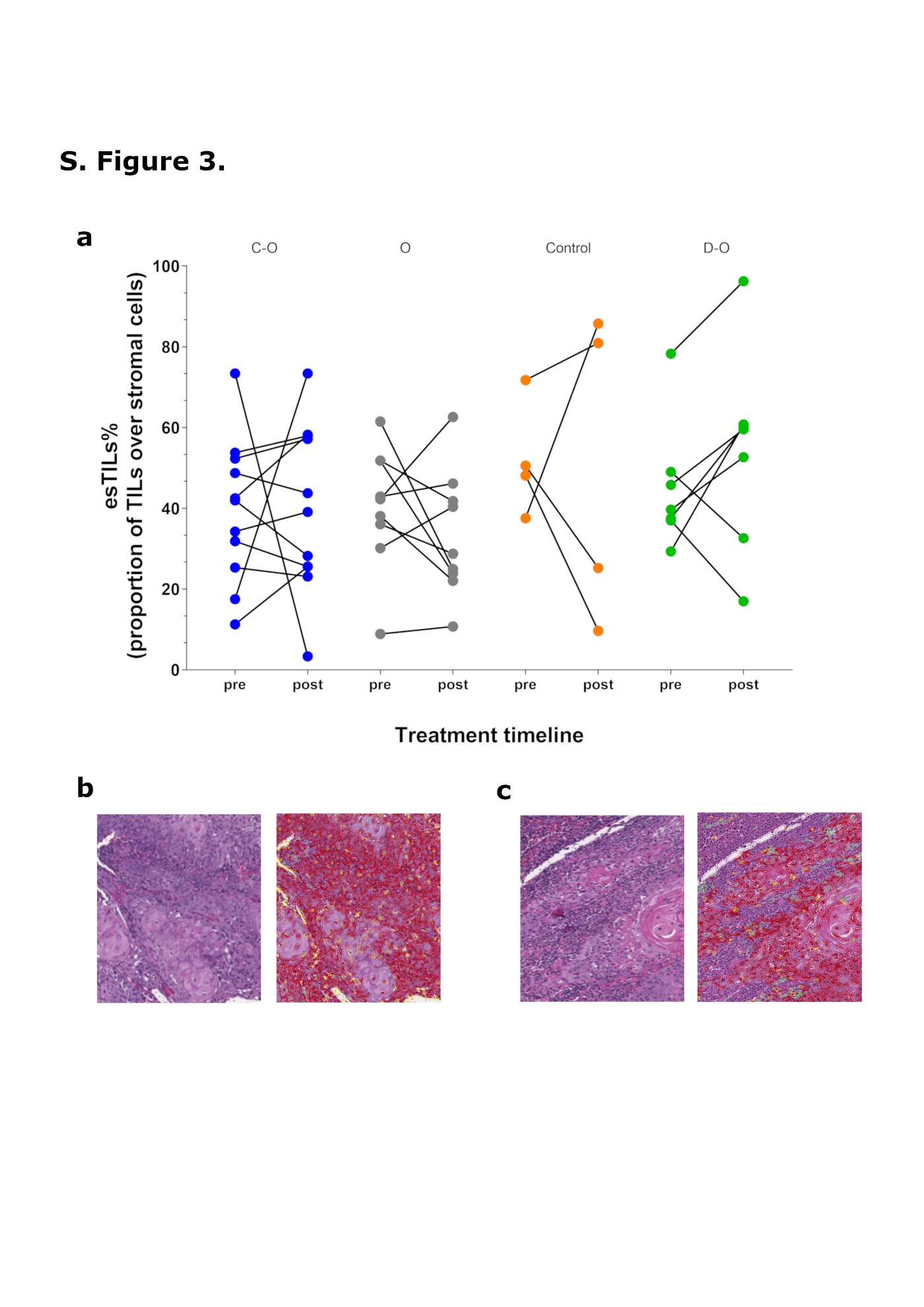

Supplement: Supplementary Figure 3 — A. No significant differences in stromal TILs were found between pre- and post-treatment samples in all arms. In Arm D TILs were increased post-treatment in most of the samples (5 out of 7). Figures B and C show representative images of TILs in pre- and post-treatment samples respectively from one patient in Arm D, measured by QuPath v0.3.0. Figures show tissues before (left) and after (right) QuPath annotations. Color legend; red, tumor cells; purple, lymphocytes; green, fibroblasts; yellow, other; Cisplatin-Olaparib, C-O; Olaparib, O; Durvalumab-Olaparib, D-O [file crc-23-0051-s09.docx]
