# Supplementary material for: Phase II Window Study of Olaparib Alone or with Cisplatin or Durvalumab in Operable Head and Neck Cancer
Source: Cancer Res Commun. 2023 Aug 10;3(8):1514–23. doi: 10.1158/2767-9764.CRC-23-0051 (PMC10414130; doi:10.1158/2767-9764.CRC-23-0051)

**Supplementary Figure 4.** Relative fold change (2^-ΔΔCq^) of *PD-L1* in respect to *Β2Μ* (reference gene) in the CTC fraction for individual samples of HNSCC patients before (blue) and after (orange) therapy.


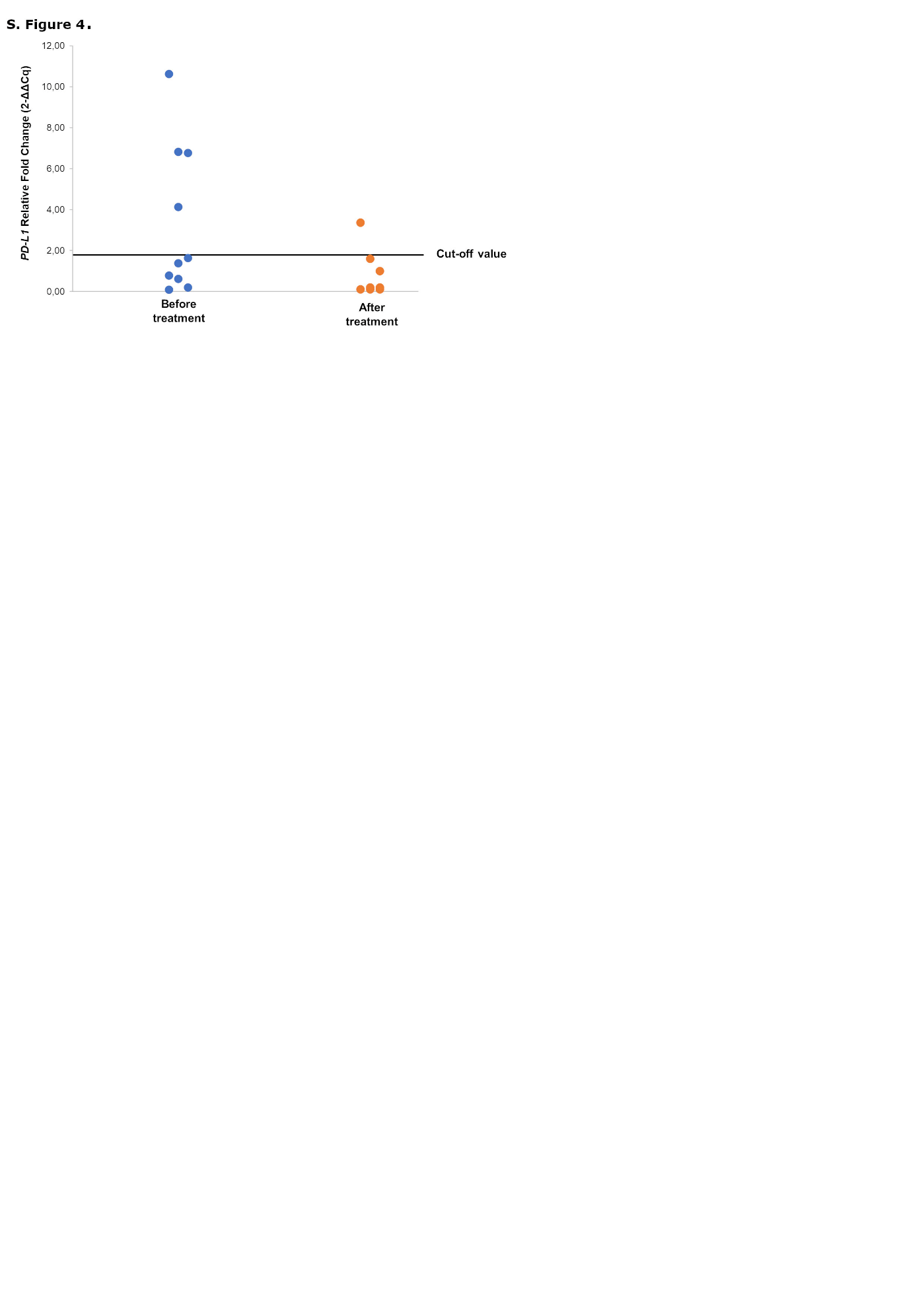

Supplement: Supplementary Figure 4 — Relative fold change (2-ΔΔCq) of PD-L1 in respect to Β2Μ (reference gene) in the CTC fraction for individual samples of HNSCC patients before (blue) and after (orange) therapy. [file crc-23-0051-s10.docx]
