# Supplementary material for: Phase II Window Study of Olaparib Alone or with Cisplatin or Durvalumab in Operable Head and Neck Cancer
Source: Cancer Res Commun. 2023 Aug 10;3(8):1514–23. doi: 10.1158/2767-9764.CRC-23-0051 (PMC10414130; doi:10.1158/2767-9764.CRC-23-0051)

**Supplementary Figure 5.** Map showing the distribution of mutations per gene per tumor. Light and dark purple, green and orange indicate pre- and post-treatment samples for patients treated with cisplatin and olaparib, olaparib and durvalumab and olaparib only, respectively. Blues correspond to samples before and after second biopsy/surgery for patients who did not receive treatment.


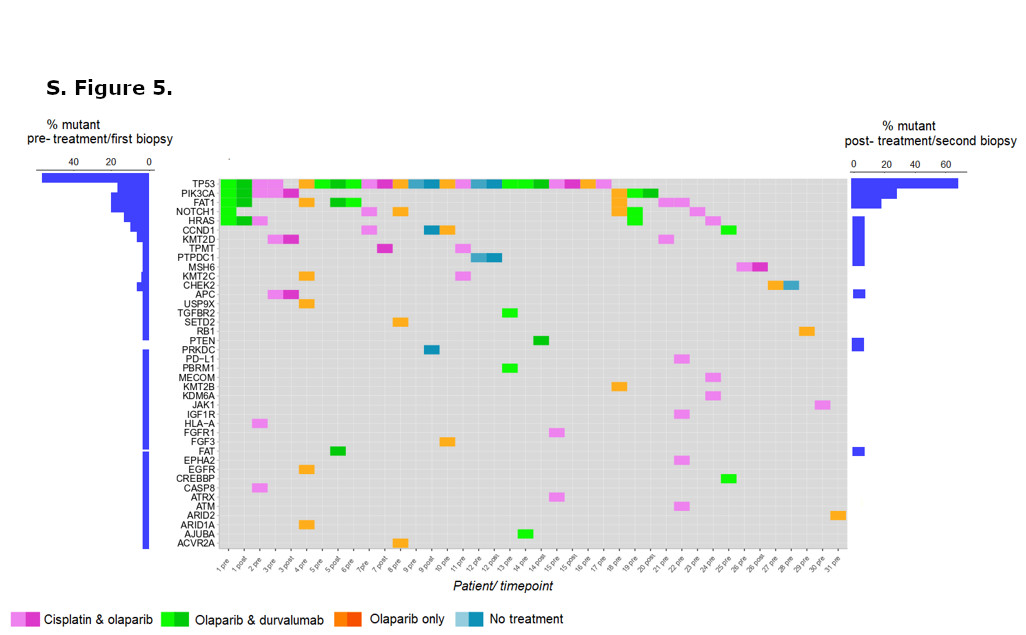

Supplement: Supplementary Figure 5 — Map showing the distribution of mutations per gene per tumor. Light and dark purple, green and orange indicate pre- and post-treatment samples for patients treated with cisplatin and olaparib, olaparib and durvalumab and olaparib only, respectively. Blues correspond to samples before and after second biopsy/surgery for patients who did not receive treatment. [file crc-23-0051-s11.docx]
