# Supplementary material for: Phase II Window Study of Olaparib Alone or with Cisplatin or Durvalumab in Operable Head and Neck Cancer
Source: Cancer Res Commun. 2023 Aug 10;3(8):1514–23. doi: 10.1158/2767-9764.CRC-23-0051 (PMC10414130; doi:10.1158/2767-9764.CRC-23-0051)

**Supplementary Figure 6.** Posttreatment CD163 (by QIF) increase in three patients with concurrent increase in the *CD163* transcripts. Representative images from a patient’s tumor tissue sample showing pre and post treatment (Durvalumab-Olaparib Arm) CD163 expression. Nuclei (blue), Cytokeratin (green), CD163 (yellow) and CSF1R (red).


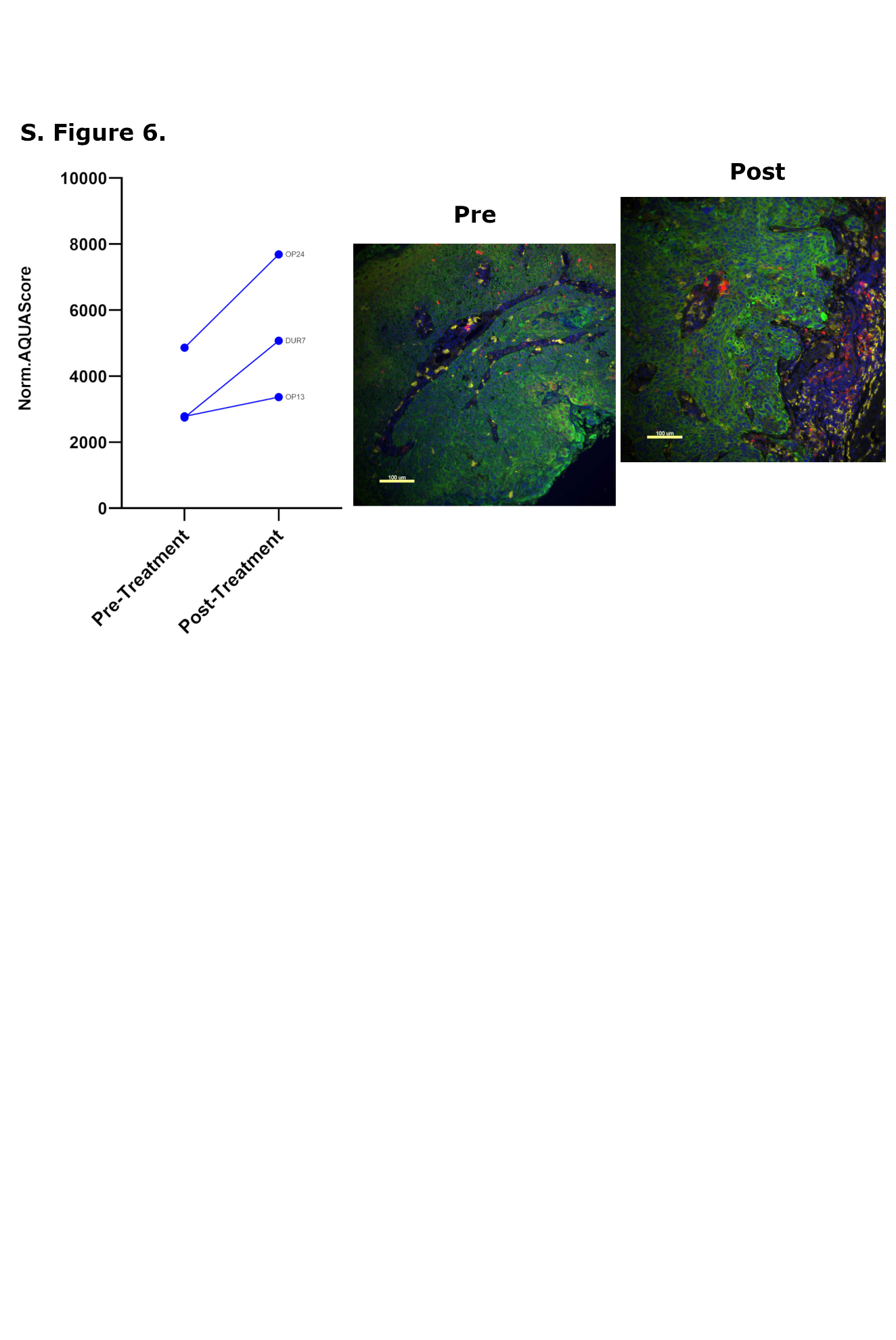

Supplement: Supplementary Figure 6 — Posttreatment CD163 (by QIF) increase in three patients with concurrent increase in the CD163 transcripts. Representative images from a patient’s tumor tissue sample showing pre and post treatment (Durvalumab-Olaparib Arm) CD163 expression. Nuclei (blue), Cytokeratin (green), CD163 (yellow) and CSF1R (red). [file crc-23-0051-s12.docx]
